# Adolescent Girls’ STEM Identity Formation and Media Images of STEM Professionals: Considering the Influence of Contextual Cues

**DOI:** 10.3389/fpsyg.2017.00716

**Published:** 2017-05-26

**Authors:** Jocelyn Steinke

**Affiliations:** School of Communication and Department of Gender and Women’s Studies, Western Michigan UniversityKalamazoo, MI, United States

**Keywords:** media images, STEM stereotypes, perceptions of scientists, science/STEM identity formation

## Abstract

Popular media have played a crucial role in the construction, representation, reproduction, and transmission of stereotypes of science, technology, engineering, and mathematics (STEM) professionals, yet little is known about how these stereotypes influence STEM identity formation. Media images of STEM professionals may be important sources of information about STEM and may be particularly salient and relevant for girls during adolescence as they actively consider future personal and professional identities. This article describes gender-stereotyped media images of STEM professionals and examines theories to identify variables that explain the potential influence of these images on STEM identity formation. Understanding these variables is important for expanding current conceptual frameworks of science/STEM identity to better determine *how* and *when* cues in the broader sociocultural context may affect adolescent girls’ STEM identity. This article emphasizes the importance of focusing on ***STEM*** ***identity relevant variables*** and ***STEM identity status*** to explain individual differences in STEM identity formation.

## Introduction

Media images of STEM (science, technology, engineering, and mathematics) professionals have varied over the years – from mad scientists to absent-minded professors to brilliant geniuses to maniacal villains to socially awkward loners to life-saving heroes. Many of these images of scientists, technologists, engineers, and mathematicians, however, have presented what has been described as a public image problem for STEM (Congressional Commission on the Advancement of Women and Minorities in Science Engineering and Technology Development, 2000). An article in *Science* noted: “Science and scientists, many observers argue, have been taking a beating in the media. The press, the movies, and especially, television convey the image that scientific progress is hazardous and that scientists are frequently foolish, inept, or even villainous” (Maugh, 1978, p. 37). An article in *New Scientist* stated that “films have always been in two minds about scientists” (Robinson, 1976, p. 732), showing real-life scientists as “humanitarian idealists” (Robinson, 1976, p. 732) and fictitious scientists as “cracked, malevolent, sometimes drunk and always dangerous” (Robinson, 1976, p. 732). An article in *The Huffington Post* explained that many stories about science in popular media often “portray scientists as falling from grace” or “fumbling and quirky, or arrogant and egotistical” and view science and technology “with fear and suspicion, more often illustrating their misuse for evil rather than their use for good” (Moreno, 2014, p. 1).

Media images of STEM professionals not only have been unflattering and unfavorable but also often have been gender stereotyped. Research shows that gender stereotypes of STEM professionals in the media influence students’ stereotyped perceptions of STEM (Steinke et al., 2007; Google-Gallup, 2015). When asked to draw or describe STEM professionals, adolescents most often depict STEM professionals as male – as well as white, middle-aged or elderly, unattractive, dressed in a lab coat and glasses, geeky or nerdy, socially awkward, and individuals who work alone (Mead and Metraux, 1957; Fort and Varney, 1989; Maoldomhnaigh and Mhaolain, 1990; Huber and Burton, 1995; She, 1995; Barman, 1996, 1999; Parsons, 1997; Song and Kim, 1999; Knight and Cunningham, 2004; Mercier et al., 2006; Scherz and Oren, 2006; Steinke et al., 2007; Buck et al., 2008; Laubach et al., 2012; Camcı-Erdoğan, 2013; Cheryan et al., 2013; Christidou et al., 2016; Sainz et al., 2016). Some variations in adolescents’ gender-stereotyped perceptions of scientists have been noted based on differences in gender (Huber and Burton, 1995; She, 1995; Steinke et al., 2007; Buck et al., 2008; Laubach et al., 2012; Hammack and High, 2014; Christidou et al., 2016), age (Mercier et al., 2006), race and ethnicity (Parsons, 1997; Finson, 2003; Monhardt, 2003; Laubach et al., 2012; O’Brien et al., 2015), and nationality (Song and Kim, 1999; Sjoberg, 2000). However, when adolescent girls view STEM fields as masculine, their perceptions can negatively affect their identification, interest, and participation in STEM (Lips, 1995; Packard and Wong, 1999; Steinke, 2003, 2005; Cheryan et al., 2015; Kessels, 2015; Carli et al., 2016). Research shows that girls first start to lose interest in STEM fields around the age of 12 (American Association of University Women, 2000) and girls report being less interested in STEM careers than boys (Riegle-Crumb et al., 2011; Robnett and Leaper, 2013; Farkas and Leaper, 2016).

Despite increases in women’s participation in some STEM fields, women still remain underrepresented in others such as economics, mathematics and statistics, computer science, engineering, and physics – fields that report disproportionate enrollment, retention, graduation, and employment rates for women (National Science Foundation, 2015). Three of four of these low participation fields have employment rates for women well below 50%, with women representing only 25.4% of all computer scientists and mathematicians, 14.8% of all engineers, and 11.8% of all physicists or astronomers (National Science Foundation, 2015). Studies focused on broadening participation in STEM have considered a variety of approaches and strategies and have identified many factors found to play a role in the underrepresentation of women in STEM (see, for example, Clewell and Campbell, 2002; Rosser, 2012). Determining the most effective strategies and best practices for recruiting and retaining women in low participation STEM fields like engineering and computer science remains a complex challenge (American Association of University Women, 2015). This issue is especially challenging because “the factors that remove women from the STEM pipeline can be seen as layers in a sex-based filter, though no single issue can be called the primary cause” (Blickenstaff, 2005, p. 384) and finding solutions to address a “complex problem like this requires a multi-faceted solution, and time to allow innovations to take effect” (Blickenstaff, 2005, p. 384).

To address the complex challenge of broadening the participation of girls and women in STEM, research and programmatic interventions have focused on an array of factors related to ***science identity*** or***STEM identity***. Research focused on the concept of science/STEM identity has been prolific in recent years, yet definitions as well as operationalizations used to assess science/STEM identity have varied widely. Additionally, variables identified as important dimensions of science/STEM identity often have been derived from research based on relatively small samples. Relatively few conceptual frameworks of science/STEM identity (Carlone and Johnson, 2007; Herrera et al., 2012) have appeared in the literature. Current conceptual frameworks of science/STEM identity (Carlone and Johnson, 2007; Herrera et al., 2012) have highlighted several critical constructs and variables for advancing understanding of science/STEM identity formation and have acknowledged environmental factors as important sources of influence. However, these frameworks have yet to fully explicate the potential influence of constructs and variables related to the broader sociocultural context such as contextual cues conveyed by popular media images of STEM professionals. Considering these constructs and variables is critical in order to better understand science/STEM identity formation for individuals with science/STEM identities in a state of flux or non-commitment.

This review examines historical trends in the presentation of media images of STEM professionals focusing on the number of female STEM professionals compared to the number of male STEM professionals and the gender stereotyped portrayals of female STEM professionals on television and in film. Next, this review draws from several theories of information processing (social cognitive or social learning theory, gender schema theory) and identity (possible selves theory, social identity theory, ego identity theory/identity status theory, and identity-based motivation theory) to identify specific constructs and variables that advance understanding of the potential influence of broader sociocultural variables on adolescent girls’ STEM identity during STEM identity formation. Finally, this review extends current conceptual frameworks of science/STEM identity by exploring *how* and *when* cultural images (as well as cultural values, cultural assumptions, cultural beliefs, and cultural stereotypes) of gender and STEM conveyed by cues in the broader sociocultural context might influence adolescent girls’ STEM identity formation.

## Media Images of STEM Professionals and Gender

Images of STEM professionals in popular media have for many years both created and perpetuated a cultural stereotype that depicts women as less likely than men to be present in STEM fields as well as less likely to be talented, successful, and valued in STEM fields. Early television programming conveyed gender stereotyped images of STEM professionals by mostly showing men as STEM professionals. Early television portrayals of scientists featured male scientists depicted as “jungle adventurers, space travelers, heroic physicians, and stereotypical ‘mad scientists”’ (LaFollette, 2013, p. 44). None of science popularizers or hosts on early television programs were women (LaFollette, 2013). This absence of women scientists on television conveyed “potent statements about the role and status of women in science” (p. 185). Although female STEM professionals have appeared more frequently in more recent television programming, the underrepresentation of female STEM professionals has persisted for years on prime-time programs broadcast between 2000 and 2008 (except for 2007) (Dudo et al., 2011), science television programs for children broadcast in 2006 and most likely to be watched by adolescents (Long et al., 2010), and some television documentaries (Hornig, 1990; Wagner and Caudill, 2003). Several more recent television programs have shown a greater number of women STEM professionals in primetime drama programs such as *CSI: Crime Scene Investigation*, *CSI: New York* and *CSI: Miami, Bones* and *Crossing Jordon* (Potter, 2008; Chandler, 2012; Warren et al., 2016) as well as some television documentaries (Steinke, 1997; Wagner and Caudill, 2003). A study of media portrayals of scientists appearing on the popular primetime television program, *The Big Bang Theory*, noted greater diversity of scientists portrayed on this program in regard to gender, ethnicity and STEM specialty, but noted that the women scientists on this program often find their work devalued or co-opted by male scientists (Weitekamp, 2015).

In addition to the relatively low numbers of female STEM professionals appearing on television, televised portrayals of female STEM professionals have often been limited and stereotyped. Female scientists appearing in children’s television science programs broadcast in 1994 typically were cast in secondary roles of lower prestige in the scientific community and shown as research assistants or students (Steinke and Long, 1996). More positive portrayals of women scientists appearing on children’s science programs were noted by a later study that found females were equally as likely as males to be labeled as scientists, to be older, and to be shown in the same status positions and noted that both female and male scientists spent equal amounts of time on screen (Long et al., 2001). A study focused specifically on television programs likely to be watched by adolescent viewers and broadcast in 2006 found depictions of male and female scientists were similar related to marital status, parental status, high status professional position; however, female scientist characters were outnumbered by male scientist characters by 2 to 1, appeared in fewer scenes, and were less likely to be shown as independent and dominant (Long et al., 2010). Another study described the infantilization and sexualization in the portrayal of Abby Sciuto, a woman scientist in the crime fiction television program *NCIS* (Bergman, 2012). A study of the primetime drama, *CSI*, noted that two of the female forensic scientists on the program, Catherine and Sara, were portrayed as making sacrifices in their personal lives for their careers, and Catherine often was portrayed in sexualized ways and depicted as an incompetent, single working mother (Warren et al., 2016). A recent study noted that two of the lead female scientists on *The Big Bang Theory*, Dr. Amy Farrah Fowler and Dr. Bernadette Wolowitz, “have been caught between their function as supporting characters for the male leads, and the potential to depict women as working scientists” (Weitekamp, 2015, p. 85).

Popular films, like television, also have perpetuated gender stereotypes of STEM professionals by showing fewer female than male STEM professionals. Studies have found that female STEM professionals have been outnumbered by male STEM professionals in 222 Hollywood fiction films across eight decades (Weingart et al., 2003), 122 film biographies of scientists (Elena, 1993), 23 popular films from 1991 to 2001 (Steinke, 2005), and films across 11 countries released in theaters from 2010 to 2013 which revealed a ratio of 7.6 STEM males to every one STEM female (Smith et al., 2014). A recent study found 42 popular films that featured female STEM professionals from 2002 to 2015 showed twice as many male STEM professionals as female STEM professionals in speaking roles (Steinke and Tavarez, 2016).

Portrayals of women as scientists, technologists, engineers, and mathematicians on the Big Screen have been diverse; however, many portrayals have focused on the appearance, femininity, and traditional gender stereotypes of female STEM professionals. Research found gender-stereotyped depictions in the 1943 film biography, *Madame Curie* (Elena, 1997), 60 feature films from 1929 to 1997 (Flicker, 2003), and 23 popular films from 1991 to 2001 that featured female scientists and engineers as primary characters (Steinke, 2005). Some progress in reducing gender-stereotyped portrayals of female STEM professionals was noted in a recent study of 62 female STEM characters in 42 popular films from 2002 to 2014 (Steinke and Tavarez, 2016). This study found more female STEM professionals were portrayed as equal contributing members of research teams and were depicted as competent although many portrayals still focused on the physical attractiveness of female STEM characters and some portrayals featured hypersexualization of these characters (Steinke and Tavarez, 2016).

## Media Framing of Gender and STEM

Media images of scientists, technologists, engineers, and mathematicians on television and in films are cultural constructions that convey assumptions about gender and STEM (Steinke, 2005). These mass-mediated messages can “be read as expressions of power in society, and as attempts to change society or mold society” (Lang, 2013). Television serves as a significant force of cultural production (Dhingra, 2006, p. 92) in American society, and television images of STEM reflect “contemporary culture on screen” (Dhingra, 2006, p. 92) “by carefully selecting the stories it tells about science and the people who tell it” (Dhingra, 2006, p. 118). Films have a similar effect: “Hollywood cinema with its perceptually realistic images and linear narrative structures contextualizes science in a manner that can establish our primary cultural meanings of science more than any other media” (Kirby, 2011, pp. 39–40). These embedded meanings have the potential to influence viewers in many ways: “The film as one of the most influential media interacts in complex ways with its audiences, reflecting, shaping, and reinforcing images and identities” (Weingart, 2006, p. 35).

Research on media frames (Gitlin, 1980; Gamson and Modigliani, 1987; Gamson et al., 1992) describes the potential of media images to shape public perceptions. Media framing research highlights the active role of the mass media in presenting “media-generated images of the world” (Gamson et al., 1992, p. 374) that intentionally construct through specific frames (Goffman, 1974) a “public identity” (van Zoonen, 1992, p. 453) of society or social issues that can shape public views of social reality when audience members encounter and interpret these images (van Zoonen, 1992). Researchers argue that through the media’s social construction of reality “[t]he lens through which we receive these images is not neutral but evinces the power and point of view of the political and economic elites who operate and focus it” (Gamson et al., 1992, p. 374). Media frames, however, represent a “mental picture of something not real or present” (Gamson et al., 1992, p. 374). Media frames also can be processed and interpreted differently by different audience members who are active processors of media content (Gamson et al., 1992). Thus, the influence of media frames varies by individual differences in audience members’ experiences and backgrounds and by their attention to and responses to what they perceive as “psychologically relevant characteristics” (Lang, 2013, p. 19) of media content.

Media frames research suggests that media images of STEM professionals offer culturally constructed views of STEM. Media images of STEM professionals are a potentially important source of “vicarious contact” (Fujioka, 1999) with STEM professionals when direct contact with real-life STEM professionals is lacking. Research has found that television, in fact, does shape adolescents’ work perceptions (Signorielli, 1993), television characters serve as professional role models that introduce adolescents to specific careers (Hoffner et al., 2006), and television characters influence adolescents’ work-related values and aspirations (Hoffner et al., 2008). Research also shows that popular media play a role in affecting how children view scientists (Tan and Jocz, 2015), and adolescents often cite popular media as sources of their images of scientists, engineers, and computer scientists (Song and Kim, 1999; Scherz and Oren, 2006; Steinke et al., 2007; Google-Gallup, 2015). Studies have found that adolescents’ exposure to media scientist characters can influence perceptions of scientists (Steinke et al., 2007) and have suggested that exposure to stereotyped media images of STEM professionals may affect interest in STEM (Lee, 1998) and influence self-identification with STEM careers (Steinke et al., 2007; Tan and Barton, 2008). Media frames research, however, points out that variations are likely to exist in adolescent girls’ individual responses to and interpretations of socially constructed media portrayals of STEM professionals.

Fictional portrayals of STEM professionals in popular media may be particularly relevant and salient sources of influence during adolescence. One researcher explains: “We can easily perceive periods in the lives of some young people where what the psychologies call ‘ego identity’ is deliberately suppressed in [favor] of socially derived norms for actions” (Solomon, 1997, p. 413). Media portrayals of STEM professionals also may be especially salient for girls during adolescence because adolescence is an active time of identity formation (Erikson, 1968). Thus, exposure throughout childhood and into the adolescent years to media portrayals that support and perpetuate a socially constructed masculine image of these fields (Weinreich-Haste, 1981; Kelly, 1985; Hughes, 2001; Blickenstaff, 2005) may elicit gender biases that directly and indirectly affect adolescent girls’ views of who belongs in STEM (see, for example, Steinke et al., 2007, 2009). Thus research in this area calls for further explication of *how* and *when* contextual cues like media images of STEM professionals are most likely to affect adolescent girls’ STEM identity formation.

## Conceptual Frameworks of Science/STEM Identity

Much research on broadening participation in STEM over the years has centered on questions related to *science identity* or perceptions of one’s self as a scientist (Hazari et al., 2010, 2013). *Identity* is defined as a “core sense of self” (Jones and McEwen, 2000, p. 405), an understanding of one’s self in relation to one’s past and potential future (Brickhouse and Potter, 2001) and “an internal, self-constructed, dynamic organization of drives, abilities, beliefs, and individual history” (Marcia, 1980, p. 159) that explain and predict behaviors (Marcia, 1994). *Identity* is dynamic (Polman and Miller, 2010; Wieland, 2010). *Identity* is malleable and changes as individuals encounter and react to events, circumstances, and elements in their social environment (Gee, 2001; Ceglie, 2011). *Identity* is both *individually constructed* (Erikson, 1968) and *socially imposed* (Marcia, 1994). *Identity* is multidimensional, involving multiple intersecting social identities (Jones and McEwen, 2000). *Identity* operates within specific contexts and structures (Herrera et al., 2012).

A science or a STEM identity has been cited as important for understanding a wide range of STEM-related outcomes including engagement, interest, learning, motivation, persistence, and commitment. Several studies have focused on the development and negotiation of science identities and science identities-in-practice for girls in both formal and informal science educational contexts (Brickhouse et al., 2000; Brickhouse and Potter, 2001; Thompson and Windschitl, 2005; Tan et al., 2013). For example, research has highlighted the importance of developing a science identity, in particular, for African–American middle school female students’ learning of science (Brickhouse et al., 2000) and for underrepresented minority students’ science career commitment (Chemers et al., 2011). Research has argued that an identity lens provides the most comprehensive understanding of STEM persistence (Carlone and Johnson, 2007; Herrera et al., 2012) and has suggested that assessing students’ science identities may be critical in predicting choice of STEM careers (Hazari et al., 2010) and encouraging student persistence in science and commitment to science careers (Trujillo and Tanner, 2014). While research on science/STEM identity has proliferated, relatively few conceptual models of science/STEM identity have appeared in the literature (Carlone and Johnson, 2007; Herrera et al., 2012).

A *science identity model* was developed by Carlone and Johnson (2007) based on their interviews with 15 women of color successful in science careers. Using an *a priori* definition to develop a prototype of a person with a strong science identity, they posited three interrelated dimensions of a science identity (competence, performance, recognition) that are influenced by one’s gender, racial, and ethnic identities (Carlone and Johnson, 2007). Based on their interviews, they noted variations in these three dimensions of science identity related to differences in science identity trajectories (research scientist, altruistic scientist, disrupted scientist) (Carlone and Johnson, 2007). They offered a grounded model of science identity that acknowledged the interactions among one’s racial, ethnic, and gender identities and the recognition dimension of a science identity (Carlone and Johnson, 2007). This model highlighted the important connection between science identity and commitment to science careers; however, this model was based on research with individuals with prior interest and a commitment to science careers and who had achieved science identities. Further exploration is needed to determine if different dimensions might be important for non-STEM identified individuals who follow different STEM identity trajectories and who are still actively exploring a STEM identity. Additional consideration also is needed of factors in non-STEM contexts that might influence the development of a STEM identity.

The *STEM identity model* developed by Herrera et al. (2012) was based on focus groups with 132 racially diverse graduate students in both STEM and non-STEM communities (Herrera et al., 2012). The STEM identity model integrates Carlone and Johnson (2007)’s model of science identity and Jones and McEwen’s (2000) model of multiple social identities with findings from their focus group interviews to explain how STEM identity interacts with three social contexts: non-STEM contexts, STEM contexts, and the societal context (Herrera et al., 2012). This model viewed STEM identity formation as including both how one perceives oneself as being recognized by others (also identified by Carlone and Johnson’s (2007) as an important dimension for achieving a science identity) in addition to how one perceives and positions oneself within STEM in light of identification with groups or communities in both STEM and non-STEM contexts situated within the larger societal context (Herrera et al., 2012). Recognition was operationalized as acknowledging historically oppressive contexts for underrepresented minority students (URM), recognizing the value of cultural knowledge, and recognizing URM students’ skills, abilities, and networks (Herrera et al., 2012). In addition, they emphasized that students’ “agency in the identity process” (Herrera et al., 2012, p. 10) and the ways students perceive themselves within STEM are just as important as recognition by others (Herrera et al., 2012). This model advanced understanding of STEM identity by specifying three different contexts that interact with STEM identity. This focus specifically highlighted the societal context as integral to understanding STEM identity. However, further explication of the potential influence of the societal context on STEM identity is needed related to how specific contextual factors affect STEM identity, the processes and pathways most likely to predict STEM identity achievement, and predictors of how and when specific factors in the societal context might influence exploration of and commitment to a STEM identity. While, this model positioned individuals as active agents in STEM identity formation and included both STEM and non-STEM individuals, further examination is needed of how sociocultural forces and change agents influence STEM identity formation for both STEM-identified and non-STEM identified individuals during intense periods of STEM identity exploration such as adolescence.

A summary of the constructs and variables of science/STEM identity described by these current conceptual frameworks are summarized in **Table 1**.

**Table 1:** Conceptual frameworks of science/STEM identity.

	Science identity model (Carlone and Johnson, 2007)	STEM identity model) Herrera et al., 2012)
Population examined	15 women of color successful in STEM careers	132 racially diverse STEM and non-STEM graduate students

Constructs and variables related to Science/STEM identity	– Competence	– Competence
	– Performance	– Performance
	– Recognition	– Recognition

Social identities considered	– Gender	– Gender
	– Racial/ethnicity	– Race/ethnicity
		– Religion/spirituality
		– Mental/physical ability
		– Socioeconomic status
		– Sexual orientation
		– Culture
		– Nationality/immigration status

STEM identity status	Achievement – professional scientists with prior interest and commitment to STEM careers	Achievement – successful STEM graduate students

Science identity trajectories	– Research scientist	None specified
	– Altruistic scientist
	– Disrupted scientist

Factors promoting Science/STEM identity	Recognition by others in scientific community	(1) Recognition by others in scientific community related to
		– URM students’ unique contribution to STEM
		– Links between URM students’ social identities and STEM identities
		(2) Self-recognition

Influence of cues in sociocultural context	Not considered	Acknowledged, but not directly examined


Both of these conceptualizations acknowledge the importance of social cues, particularly those in STEM environments, related to recognition as a scientist in influencing science/STEM identity (Carlone and Johnson, 2007; Herrera et al., 2012). Carlone and Johnson (2007) specifically noted challenges posed by the “institutional and historical meanings of being a scientist (being a white male)” (p. 1207) in achieving the recognition dimension of a science identity for women of color: “It is much easier to get recognized as a scientist if your ways of talking, looking, acting and interacting align with historical and prototypical notions of scientist” (p. 1207). Similarly, Herrera et al. (2012) emphasized that identity operates within contexts, highlighting the role of societal context, with its underlying social norms, social expectations, and social roles, on STEM identity development and enactment. Further explication is needed of the potential influence of contextual cues in the broader sociocultural context that are first experienced during the early years of childhood and throughout adolescence.

Extant conceptual frameworks of science/STEM identity have highlighted many important constructs and variables related to STEM identity; however, explanations of *how* and *when* variables like contextual cues in the broader sociocultural context might affect STEM identity formation have been left relatively unexplored. The following section draws from information processing theories and identity theories to consider the potential influence of broader cues in the sociocultural context, such as cues conveyed through media portrayals of STEM professionals. Little is known about *how* and *when* these contextual cues may affect STEM identity, and in particular, how they may affect STEM identity during active periods of STEM identity exploration. Understanding this issue has practical importance because of the continued underrepresentation of girls and women in STEM and the multitude of programs and interventions focused on broadening STEM participation. In order to better explain and predict the potential influence of contextual cues conveyed through media portrayals of STEM professionals on adolescent girls’ STEM identity formation, several theories of information processing and identity from various disciplines are presented below. Collectively, these theories (1) advance understanding of *how* and *when* specific characteristics of media models affect adolescent girls’ STEM identity formation and (2) *how* and *when* variations in STEM identity status and identity processing lead to differences in the influence of media models on adolescent girls during STEM identity formation.

## Theoretical Perspectives on Processing Gender Stereotypes of STEM

As previously described in this review, research has examined gender-stereotyped portrayals of STEM professionals appearing in popular media and has considered the potential influence of these media images on adolescent girls’ perceptions of STEM professionals and STEM careers. Few studies, however, have examined specifically *how* and *when* these media images are most likely to exert influence or have considered *individual differences* in audience members’ processing of these media images. Several theories of information processing and identity are described below in order to better explain the potential influence of media images of STEM professionals on adolescent girls’ STEM identity formation.

### Social Cognitive (Learning) Theory

Social cognitive theory, formerly social learning theory, explains *how* stereotypes in the media influence individuals’ attitudes and behaviors. According to this theory, repeated observation of both actual models as well as symbolic models teaches cultural patterns of behaviors (Bandura, 1969, 1986). Social learning theory describes how children learn to imitate behaviors from others in their environments through the process of “identificatory learning” (Bandura et al., 1963, p. 533). Research dating back to the 1950s showed that viewers identified with film characters of the same sex and more accurately remembered the words and actions of characters with whom they identified (Maccoby and Wilson, 1957). This research also found that women were more likely than men to remember female characters who they perceived as important and more likely to remember the actions of female characters shown in romantic scenes (Maccoby and Wilson, 1957).

Social cognitive theory contributes to a broader understanding of *how* gender stereotypes of STEM professionals in popular media may affect adolescent girls’ STEM identity formation in several important ways. First, it explains how vicarious exposure to media characters can lead to modeling the behavior of STEM characters in the media thereby influencing not only perceptions but also behavior. Second, social cognitive theory describes differences in the influence of media models based on specific characteristics or attributes of media models, such as the sex or other attributes of the media model. Social cognitive theory also suggests gender differences in how viewers are influenced by media models. Third, social cognitive theory indicates that individuals’ identification with media models affects the extent of influence on individuals. *Thus, social cognitive theory suggests that media images of STEM professionals can be important models for adolescent girls and are most likely to influence adolescent girls when they share characteristics and traits perceived as similar or desirable by adolescent girls.*

### Gender Schema Theory

Gender schema theory explains *how* networks of knowledge about gender, learned in part through identificatory learning, are developed and activated in children’s memories (Bem, 1981). These networks or gender schemas are “cognitive structures that organize gender-related knowledge, beliefs, attitudes, and preferences” (Liben and Signorella, 1993). Gender schemas, just like other schemas, help children understand experiences and make decisions that influence their perceptions, beliefs, and behavior (Bem, 1993). Research suggests that portrayals of characters on television programs can shape and maintain gender schemas (Leaper et al., 2002), particularly if children identify with those characters (Miller and Reeves, 1976).

Gender schema theory contributes to a broader understanding of *how* and *when* gender stereotypes of STEM professionals in popular media may affect adolescent girls’ STEM identity formation by emphasizing the dominant role of gender schemas during information processing. In societal contexts where gender is made salient (i.e., watching television programs or films that show images of female STEM professionals or that stereotype female STEM professionals), gender schemas may be more likely to be activated, becoming especially salient, dominant, important, and/or influential. One study noted that subtle contextual cues of incompatibility between gender and STEM, such as video games or posters or sci-fi movies that depict traditionally male interests, may pose a threat to gender-STEM compatibility (Cheryan et al., 2009). *Thus, gender schema theory suggests that female STEM professional media models may be especially salient for adolescent girls when they prioritize the processing of information about and from these models based on gender or when their gender schemas are activated.*

### Social Identity Theory

Social identity theory explains *how* individuals negotiate and form an identity through multiple affiliations and disassociations with various social groups (Tajfel, 1978, 1982; Tajfel and Turner, 1979) as they strive to maintain a positive social identity through affiliation with in-groups rather than out-groups (Tajfel and Turner, 1979). According to this theory, “[s]ocial structures (e.g., of gender, class, ‘race’) thus play an important role in shaping the identities, choices, and aspirations that people perceive as possible and desirable” (Archer et al., 2012, p. 970). Individuals develop identity schemas, cognitive structures of “complex, rich, affectively charged, interrelated concepts about the self” (Korte, 2007, p. 168), that serve as “internal organizations of stored information and meanings operating as frameworks for interpreting experiences” (Stryker and Serpe, 1994, p. 18).

Social identity theory contributes to a broader understanding of *how* gender stereotypes of STEM professionals in the media may affect adolescent girls’ STEM identity formation in a couple of important ways. First, social identity theory highlights the active role of individuals in maintaining varied social or group identities or in-group affiliations they most support (Burke and Reitzes, 1991; Stets and Burke, 2000). As adolescent girls contemplate their future careers, they actively negotiate multiple identities that include personal relationships, family roles, and cultural values (Thompson and Windschitl, 2005). Second, it acknowledges the importance and centrality of those particular identities to which an individual holds a strong commitment (Burke, 1991; Burke and Reitzes, 1991). Research shows that a central identity may change over time (Settles et al., 2009) and identity interference can occur from conflicting identities (Settles, 2004; Ahlqvist et al., 2013). *Social identity theory suggests that girls are most likely to identify with media models of female STEM professionals who they perceive belong to similar and/or desirable in-groups (i.e., gender, race, ethnicity, and other in-groups). This theory also suggest that girls are most likely to identify with media models of female STEM professionals when they view both themselves and females as part of or belonging to a STEM in-group. Additionally, this theory suggests that gender-stereotyped media models of STEM professionals may elicit identity interference for adolescent girls if exposure to these model leads them to perceive that a female gender identity and a STEM identity are incompatible.*

### Possible Selves Theory

Possible selves theory describes *how* current and future representations of who one is and who one might become motivate behavior (Markus and Nurius, 1986) and “translate into life choices” (Lips, 2007, p. 51). According to this theory, an individual’s self-concept “is viewed as a system of affective-cognitive structures or schemas about one’s self” (Markus and Nurius, 1986, p. 955). Self-concepts include an individual’s perceptions of their possible selves (Markus and Nurius, 1986). Possible selves correspond to both current representations of who one is as well hypothetical images of who one might become in the future (Strahan and Wilson, 2006). Possible selves present both hoped for and feared representations of self (Markus and Nurius, 1986). Possible selves are influential because they motivate behavior related to life goals (Markus and Nurius, 1986; Strahan and Wilson, 2006), such as academic achievement (Oyserman et al., 2004), career aspirations (Markus and Nurius, 1986), and future career selection (Lips, 2007). Research also has found that possible selves and strategies to attain particular possible selves are sensitive to contextual cues that can make stereotypes more accessible and salient (Bi and Oyserman, 2015).

Possible selves theory contributes to a broader understanding of *how* gender stereotypes of STEM professionals in the media may affect adolescent girls STEM identity formation in a couple of ways. First, it highlights how media models might inspire both current and future representations of one’s self. Second, it emphasizes that media models representing hoped-for future selves can motivate behavior related to career aspirations. Adolescent girls’ development of possible selves based on cultural representations of gender conveyed by media portrayals of STEM professionals also may motivate behavior related to pursing STEM careers and may affect STEM identity. *Possible selves theory suggest that gender-stereotyped media models of STEM professionals present images of potential, possible selves. The ways female STEM professionals are portrayed by media models may influence whether adolescent girls view these models as hoped-for or feared-possible selves and whether they inspire to a future career in STEM.*

### Ego Identity Formation Theory and Identity Status

Ego identity formation theory emphasizes *when* gender stereotypes of STEM professionals in the media are most likely to affect adolescent girls’ STEM identity formation. Ego identity formation theory describes the psychological processing or *exploration* of particular components of identity (gender, race, ethnicity, etc.) that eventually leads to commitment to a specific component identity (Erikson, 1968; Umana-Taylor et al., 2004). Erikson (1968) describes identity formation as a mostly unconscious “process of simultaneous reflection and observation, a processing taking place on all levels of mental function” (p. 22). Research shows that conceptions of identities are always changing because they are reflexive (Stets and Burke, 2000), dynamic (Wieland, 2010; Ahlqvist et al., 2013) and not “just states or traits of an individual that are relatively fixed” (Burke and Reitzes, 1991, p. 847). Identity is developed through exploration and commitment across a variety of identity domains, including vocational or occupational choice (Erikson, 1968; Umana-Taylor et al., 2004).

Extending Erikson’s theory, Marcia (1994) classified individuals according to one of four identity statuses based on the degree of *exploration and commitment* (Marcia, 1994) during decision making. *Moratorium identity status* describes those who have explored but not yet committed to an identity (Marcia, 1994). *Diffuse identity status* describes those who have not yet explored and not committed to an identity (Marcia, 1994). *Foreclosed identity status* describes those who have not explored, instead accepting others’ choices as their own, and have committed to an identity (Marcia, 1994). *Achieved identity status* describes those who have both explored and committed to an identity (Marcia, 1994).

**FIGURE 1:**
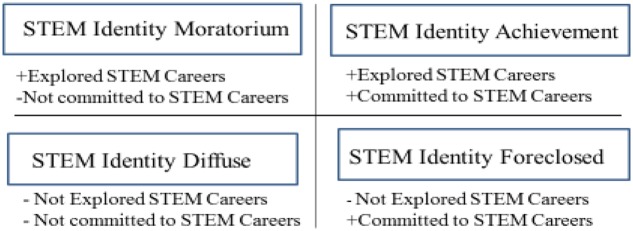
**Science identity status based on Marcia’s (1980) identity status theory**.

Ego identity theory and identity status theory contribute to a broader understanding of *when* gender stereotypes of STEM professionals in the media may affect adolescent girls STEM identity development in two important ways. First, these theoretical perspectives acknowledge an individual’s active consideration of a STEM identity based on the degree of exploration and commitment to that identity. Second, these theoretical perspectives outline two critical conditions (STEM exploration plus STEM commitment) needed for achievement of a STEM identity (**Figure 1**). *Ego identity theory and identity status theory suggest that gender-stereotyped media models of STEM professionals are most likely to be influential for adolescent girls who are actively exploring a STEM identity and may not be as influential for adolescent girls who have already committed to a STEM identity.*

### Identity-Based Motivation Theory

Identity-based motivation theory describes both *how* and *when* people’s current and active identities motivate their behavior based on the ways in which contextual cues actively shape identity in a particular context (Oyserman, 2014, 2015a). According to identity-based motivation theory, (1) contextual cues trigger identities that are dynamically constructed and determine which personal and social identities are perceived as most accessible in that moment, (2) accessible identities influence action-readiness based on perceived necessity and relevance, and (3) activated, accessible identities influence behavior depending on perceptions of difficulty (Oyserman, 2014, 2015a,b). The premise of identity-based motivation theory is that “thinking is influenced by the context in which it occurs” (Oyserman, 2015a, p. 2). Identity-based motivation theory focuses on the underlying process for determining the circumstances in which aspects of identity matter for behavior perceived to be difficult (Oyserman, 2015b).

Identity-based motivation theory contributes to a broader understanding of both *how* and *when* gender stereotypes of STEM professionals in the media may affect adolescent girls STEM identity formation in a number of important ways. First, it highlights the dynamic, changing nature of identity. Second, it considers how contextual cues – described as “features in the environment” (Oyserman, 2015a, p. 2) – influence identity and directly trigger “what comes to mind when people consider who they are” (Oyserman, 2015a, p. 2). Third, it describes how specific identities are accessed and then influence action based on perceptions of necessity and relevance. Fourth, it explains how activated identities motivate behavior depending on perceptions of how difficult the behavior is (Oyserman, 2015b). *Identity-based motivation theory provides a more comprehensive and considered approach to understanding the potential influence of gender- stereotyped media models of STEM professionals on adolescent girls’ STEM identity. Specifically, this theory suggests that media models are most likely to influence adolescent girls who are actively exploring a STEM identity and are likely to exert influence when they trigger or make accessible a STEM identity and when adolescent girls perceive behaviors related to achieving a STEM identity as necessary and relevant to them and not too difficult to achieve.*

A summary of potential media model effects on identity formation as described by these theories are presented in **Table 2**.

**Table 2:** Theories describing potential model influence related to identity formation.

	Social cognitive theory	Gender schema theory	Social identity theory	Possible selves theory	Ego-identity theory/identity status theory	Identity-based motivation theory
Traits and attributes of models	Vicarious models, media models	Same-sex models	Same-social identity group models	Possible-self-models	__	Same-social identities models

Identity relevant characteristics of models	Similar; important; or desirable	Gender-compatible	In-group compatible	Hoped-for self, both current and future	__	Accessible and attainable (not too difficult)

Conditions for model influence	__	__	__	__	Degree of exploration and commitment to identity	Identity triggered by contextual cues; accessible; attainable

Type of model influence explained	How	How and When	How	How	When	How and When


The theories described above identify additional constructs and variables important for understanding *how* and *when* images of STEM professionals in popular media are most likely to influence STEM identity formation for adolescent girls. In order to better understand the influence of gender-stereotyped media images of STEM professionals on STEM identity, it is important to consider the ***STEM identity relevant variables*** perceived by adolescent girls and the ***STEM identity status*** of adolescent girls. As these theories have highlighted, the influence of media models of STEM professionals on adolescent girls’ STEM identity will vary depending on (1) differences in media model characteristics, (2) differences in adolescent girls’ perceptions of the psychological relevance of different traits and attributes of the models, (3) differences in adolescent girls’ STEM identity status (degree of exploration and commitment), and (4) differences in adolescent girls’ accessibility to a STEM identity and perceived attainability of behaviors related to STEM identity achievement. The implications for future theory development and research in this area are described in the section that follows.

## Extending Conceptualizations of STEM Identity

Extant theories of information processing and identity offer useful insights for extending current conceptualizations of science/STEM identity. Current conceptualizations have identified several individual, social, and contextual factors that influence STEM identity (Carlone and Johnson, 2007; Herrera et al., 2012). However, extending these conceptual frameworks to include variables of potential influence related to the broader sociocultural context will further advance understanding of how gender-stereotyped images of STEM professionals can influence adolescent girls’ STEM identity formation. Specifically, consideration of these variables highlights the importance of recognizing individual differences in perceptions of ***STEM*** ***identity relevant variables*** as well as individual differences in***STEM identity status***. Drawing additional constructs and variables from the theories described above to extend current theoretical conceptualization of STEM identity not only helps better predict *how* and *when* contextual cues, including gender stereotypes, affect STEM identity achievement but also highlights the dynamic nature of STEM identity that is particularly pronounced during adolescence when STEM identity exploration is especially active. This extension of current conceptualizations of science/STEM identity has important implications for future research and practice.

The constructs and variables derived from extant theories of information processing and identity outlined in this review help provide a more comprehensive and nuanced conceptualization of STEM identity. As the theories described above suggest, understanding the potential influence of cultural representations of gender and STEM may potentially be particularly relevant for adolescent girls who frequently encounter gender-stereotyped media images of STEM professionals, who perceive these images to be salient, who perceive being female and STEM to be incompatible social identities, who have spent less time on STEM identity exploration, and who have not yet committed to a STEM identity. The section that follow discusses specific constructs drawn from the theories reviewed above and represents an initial step toward re-conceptualizing STEM identity to more fully develop an understanding of the potential influence of contextual cues on adolescent girls’ STEM identity formation.

### Differences in Media Model Characteristics

The creation of more and more diverse media models of female STEM professionals may be critical for challenging and changing the dominant masculine image of STEM professionals. Research has shown that female role models can have a positive impact, in particular, on adolescent girls’ attitudes toward science (Evans et al., 1995) and their stereotyped perceptions of STEM professionals (Hughes et al., 2013). One study that focused on the influence of diverse, female role models for non-STEM-identified adolescent girls noted signs of more positive endorsement of a STEM identity, which was indirectly assessed based on changes in measures of STEM interest and science and math self-concepts (Hughes et al., 2013). While this research focused exclusively on the impact of real-life female role models in a STEM environment, specifically a STEM camp (Hughes et al., 2013), additional research needs to assess individual differences in identification with a diverse array of media models and examine how individual differences in identities, including differences in identity centrality for multiple salient identities, affect identification with media models. It is important to extend research that has argued for “matched role models” (Gilmartin et al., 2007, p. 984) and consider which specific characteristics of are models are most “psychologically relevant” (Lang, 2013) for adolescent girls. Future research should focus on the ***identity relevant characteristics*** of media models of female STEM professionals to further understanding of most effective strategies and interventions for promoting a STEM identity.

Extensions of conceptual framework of STEM identity to include the influence of contextual cues from media models would provide a broader understanding of the greater array of contextual cues on STEM identity formation. Media models provide “vicarious contact” (Fujioka, 1999) with STEM professionals when direct contact with real-life STEM professionals is lacking. However, little is known about how inspiring media images of STEM professionals that deviate from the prevailing stereotypical media images of STEM professionals might act as important role models for adolescent girls during science identity formation. Several theories of the theories reviewed above (social learning theory, gender schema theory, social identity theory) emphasize that specific attributes or characteristics of media models are important for assessing influences on STEM identity. Thus, future research focused on carefully controlled studies of specific media model attributes or characteristics (gender, race/ethnicity, perceived similarity, perceived desirability) and direct measures of STEM identity are important for advancing understanding of the potential influence of media models of STEM professionals on adolescent girls’ STEM identity achievement.

### Differences in Psychological Relevance of Media Model Characteristics

Existing conceptual frameworks of science/STEM identity recognize the importance of integrating a science/STEM identity with other social identities (Carlone and Johnson, 2007; Herrera et al., 2012). In seeking ways to promote the formation of a STEM identity for students, it is important to know how they “are engaging in science and how this is related to who they think they are (what communities of practice the participate in), e.g., good student, a basketball player, a gossip, and who they want to be (what communities practice they aspire to), e.g., a teacher, a mother, a gemologist, an obstetrician” (Brickhouse et al., 2000, p. 443). This research suggests that the social identities of media models matter and also are likely to influence STEM identity formation. Future research focused on a wider array of characteristics of media models related to their social identities is needed to determine which characteristics are most salient and most likely to affect change in STEM identity for adolescent girls.

Possible selves theories highlights unique differences in individuals’ processing of information about possible identities in light of hoped for future selves and future goals. Additional research should not only examine which characteristics of media models of STEM professionals are most salient, but also which characteristics are most salient for different individuals with different future life and career goals. Differences related to individuals’ backgrounds and experiences also need to be considered in order to describe variations in processing, interpretations, and effects of various media model characteristics. Communication scholar Annie Lang has argued for research that takes into account the “psychologically relevant characteristics” (Lang, 2013, p. 19) of media content. Advancing conceptual understanding of the influence of contextual cues on STEM identity will require research that considers the psychological relevance of a diverse array of characteristics of media models related to the ***STEM identity relevant variables*** of the media models.

### Differences in STEM Identity Status and Potential Influence of Media Models

Current conceptual frameworks of science/STEM identity have focused primarily on individuals *committed to or in the process of committing* to a STEM identity (Carlone and Johnson, 2007; Herrera et al., 2012). This focus on STEM-identified individuals has been helpful in identifying factors, such as recognition by others in the scientific community and recognition of other social identities, found to be important for the achievement of a STEM identity. Extending this work by considering how non-STEM-identified individual currently *exploring* and who have yet to commit to a STEM identity is needed to determine factors that impede or hinder the achievement of a STEM identity. *Science identity* has been described as an “evolving construct” that “falls along a continuum – or multiple continuums, different for different groups of students with different sets of experiences and contexts” (Gilmartin et al., 2007, p. 982). However, relatively few studies have considered science/STEM identity as a continuous variable based on individual differences in the degree of exploration and commitment to a STEM identity or individual differences in STEM identity status (Perez et al., 2013). This approach is particularly important when assessing STEM identity formation for non-STEM identified (or non-STEM committed) individuals and for individuals like adolescents who are undergoing a particularly active period of identity exploration.

Ego identity theory and identity status theory explain that individuals exhibit one of four possible STEM identity statuses depending on their exploration of and commitment to a STEM identity (**Figure 1**). Future research assessing the influence of contextual cues on STEM identity formation need to focus on the dynamic, fluctuating nature of identity and specifically how differences in the degree of STEM identity exploration and commitment moderate the potential influence of these contextual cues. STEM-committed compared to non-STEM-committed adolescent girls may be less likely to be influenced by gendered stereotyped media images of STEM professionals. Future research needs to assess STEM identity status and differences in STEM identity status and how it varies over time using longitudinal studies that advance understanding of changes in STEM identity during different developmental stages.

### Differences in Accessing and Perceptions of Attainment and Difficulty of a STEM Identity

Current conceptual frameworks of science/STEM identity have focused on identifying specific dimensions of STEM identity and specific factors related to STEM identity formation (Carlone and Johnson, 2007; Herrera et al., 2012) but have left relatively unexplored individuals’ management of a STEM identity and the ways in which STEM identity motivates STEM-related behaviors like taking STEM courses, selecting a STEM major, and choosing a STEM career. A better understanding of the motivations for engaging in identity work will help better predict how a STEM identity is enacted.

Identity-based motivation theory describes how identity can motivate such behavior. Identity-based motivation theory suggests how media models might trigger or make accessible a STEM identity – and influence their impact (perceived attainability, perceived difficulty) in motivating behavior during STEM identity formation. Future research needs to focus on how contextual cues like media models trigger a STEM identity and influence perceptions of attainability and difficulty of STEM-related behaviors for adolescent girls.

Research dating back to the mid-1990s has focused on science or STEM identity as critical for broadening participation in STEM (for a review of research published between 1995 and 2006, see Brotman and Moore, 2008). Additional meta analyses of this literature and additional testing of constructs and variables identified from existing theories as well as new research related to other constructs and variables that may influence STEM identity will do much to advance current conceptualizations of STEM identity. Further consideration of how to define the concept of science/STEM identity is needed and will facilitate the development and use of more direct and consistent operationalizations to assess science/STEM identity. Current conceptualizations of science/STEM identity position science/STEM identity as a collective construct for all science or STEM disciplines; however, STEM identity formation may not necessarily involve the same constructs and variables, follow the same process, or manifest identity status in the same ways for each specific STEM discipline (see, for example, Hazari et al., 2010 for an explication of a physics identity framework that adds *interest* as an additional construct or variable). It may be necessary to consider a multitude of identity statuses that vary by science identity, technology identity, engineering identity, and mathematics identity and consider the degree of exploration and commitment for each of these – individual – STEM identities. Specifically, a STEM identity may not be the same across all STEM fields. Identity as related to the array of possible and multiple STEM identities may differ in several important yet to be determined ways.

## Conclusion

Expanding current conceptualizations of STEM identity to consider individual differences in perceptions of ***STEM identity relevant variables*** and by ***STEM identity status*** (the degree of exploration and commitment to a STEM identity) offers promising directions to advance theory, research, and practice related to broadening the participation of girls and women in STEM. Closing the gender gap in low participation STEM fields is critical not only for achieving equity in society (Laughter and Adams, 2012) but also for advancing innovative ideas and approaches needed to ensure future economic progress and competitiveness (Rosser, 2012). An array of factors at home, in schools, and in media contribute many different constructs and variables that collectively may have different effects at different times and in different contexts for individuals of different backgrounds, experiences, perspectives, personalities, and identities. Expanding current conceptualizations of science/STEM identity to consider *how* and *when* contextual cues in the sociocultural environment affect STEM identity may be particularly useful for developing a more comprehensive and nuanced approach to understanding STEM identity.

Additional theoretical reconceptualization of STEM identity and future research focused on a wider array of relevant contexts in which STEM identity work is enacted will aid in developing a more comprehensive theoretical framework in several ways. First, additional research employing multiple and varied methodologies and longitudinal studies will provide insights about the many constructs and variables at the individual, social, and broader sociocultural levels as well as at the intersections of these different levels of analysis. Second, research that considers variations related to ***STEM identity relevant variables*** will help determine the most salient variables related to STEM identity for various populations. Third, in order to advance understanding of the dynamic nature of STEM identity formation, future research focused on variations in ***STEM identity status*** will help determine differences in the influence of constructs and variables based on fluctuations in STEM identity. Collectively, this research will help determine best practices and the most effective interventions for broadening the participation of girls and young women in a variety of different contexts related to STEM.

This review draws from several extant theories of information processing and identity to describe the potential influence of cues from the sociocultural context (i.e., gender stereotypes of STEM professionals) on STEM identity formation. Greater consideration of *how* and *when* contextual cues conveyed through images of STEM professionals in popular media might affect STEM identity formation offers new insights to extend existing conceptual theoretical frameworks of science/STEM identity and advance understanding of STEM identity formation for adolescent girls. Identifying factors that help explain how long-held, gender stereotypes of STEM professionals derived from popular media may shape STEM identity can better inform effective interventions specifically those intended for non-STEM identified adolescent girls. Researchers have argued that how girls “describe personal relevance when they are engaged across different contexts relevant to their identities” (Thompson and Windschitl, 2005, p. 18) is dependent on their (1) perceptions of who they are, (2) who they are becoming in the future, and (3) personal relationships with family and friends (Thompson and Windschitl, 2005). Future theoretically driven research in this area may be especially important for developing a deeper understanding of adolescent girls’ STEM identity formation when they encounter contextual cues transmitted by popular media that promote a masculine image of STEM professionals who adolescent girls dismiss as not relevant and describe as just “not like me” (MacDonald, 2014).

## Author Contributions

The author confirms being the sole contributor of this work and approved it for publication.

## Conflict of Interest Statement

The author declares that the research was conducted in the absence of any commercial or financial relationships that could be construed as a potential conflict of interest.
